# Patient Satisfaction with a Novel Tele-Cystoscopy Model: Expanding Access to Bladder Cancer Surveillance for Rural Patients

**DOI:** 10.1089/tmr.2024.0034

**Published:** 2024-08-05

**Authors:** Aisha O. Kazeem, William Hasken, Terran Sims, Stephen H. Culp, Tracey L. Krupski, Jennifer M. Lobo

**Affiliations:** ^1^ Department of Urology, University of Virginia, Charlottesville, Virginia, USA.; ^2^ University of Virginia School of Medicine, Charlottesville, Virginia, USA.; ^3^ Department of Public Health Sciences, School of Medicine, University of Virginia, Charlottesville, Virginia, USA.

**Keywords:** cystoscopy, rural health, telemedicine, videoconferencing

## Abstract

**Background:**

Tele-cystoscopy involves trained advanced practice providers performing cystoscopy with real-time interpretation by an urologist. The goal of this externally validated care model is to expand the availability of cystoscopy to underserved rural areas. Herein we report on population demographics and describe the socioeconomic benefits of tele-cystoscopy for bladder cancer surveillance.

**Methods:**

Using an IRB-approved protocol, patients were consented for dual, sequential cystoscopy wherein they experienced a standard-of-care cystoscopy along with tele-cystoscopy. Patients completed a questionnaire that contained both subjective and objective health and socioeconomic-related questions as well as a satisfaction survey. Patients were also probed about factors associated with transportation to their cystoscopy appointments including gasoline costs, travel time, and time off work. Using the Distressed Community Index, patients were ascribed an economic resource category ranging from prosperous to distressed.

**Results:**

In total, 48 patients with a mean age of 55 completed surveys after completing dual cystoscopies. Thirteen patients (27%) were uninsured and 10 patients (20%) had Medicaid as primary insurance. The tele-cystoscopy clinic saved patients an average of 235 miles and 434 min of travel time. In total, 82% of patients resided in a distressed community indicating fewer economic resources. Satisfaction results showed a mean score of 31.38 (out of 32).

**Conclusions:**

Patients were satisfied with tele-cystoscopy, noting increased access to health care and fewer disruptions impacting bladder cancer surveillance. Tele-cystoscopy may be a viable option to expand access and improve adherence to guidelines for bladder cancer surveillance, particularly benefiting patients in rural areas and those of lower socioeconomic status.

## Introduction

The field of urology faces extreme workforce shortages in the near future. Although the overall number of urologists has grown since the 1980s, the supply of urologists has declined per capita compared to other surgical subspecialties such as neurosurgery, otolaryngology, and orthopedics.^1^ Indeed, even with the relative increase in resident training positions and the addition of accredited urology residency programs, another projection found that the number of urologists per capita will reach a trough of 15.8 urologists per 100,000 persons 65 years and older in 2035.^2^ A review in 2022 of the American Urological Association (AUA) census data revealed that rural communities will be the most impacted by the urology shortage.^3^

The utilization of telemedicine and incorporation of advanced practice providers (APPs) are two oft-cited ways of increasing rural access to urologic care and managing urology workforce shortages.^1,4–9^ The AUA has reported that overall, 71.5% of urologists used telemedicine and 81.5% of urologists reported working with at least one APP in their practice. Notably, the use of APPs among rural urologists was lower at 62%, perhaps indicating a difference in practice structure as rural urologists are more likely to be solo practitioners.^4,10,11^

### Bladder cancer diagnosis and surveillance

Bladder cancer is the 10th most common cancer worldwide and has increasing incidence in developing nations. If detected at early stages, nonmuscle invasive bladder cancer (NMBIC) is treated with transurethral resection and intravesical therapies. Current AUA guidelines recommend surveillance cystoscopy at varying intervals depending on a patient’s risk for progression or recurrence.^12^ The uneven distribution of urologists at a county level has been shown to have an impact on urologic cancer mortality, including bladder cancer. Odisho et al. noted that counties with >0–2 urologists per 100,000 people had an 18% lower mortality rate than counties with no urologists and that metropolitan counties had a 9% lower mortality rate than nonmetropolitan counties.^13^ A 2017 study found bladder cancer mortality ranging from 2.5 to 8.9 deaths per 100,000 people.^14^ This disparity between rural and urban areas was highlighted at the American Society of Clinical Oncology meeting in 2024 when authors from Cleveland Clinic found significant improvement in bladder cancer mortality rates for urban areas but not for rural areas over the decades from 2002 to 2020.^15^ Cystoscopy performed by a urologist or urology-trained APP is the centerpiece of bladder cancer diagnosis and surveillance for recurrence.^16^ Though a telemedicine visit cannot replace cystoscopy, our previous research has shown that APP-led tele-cystoscopy models are a viable solution for accessing bladder cancer care. Early identification of bladder cancer is the first step in improving survival rates.

Barriers to rural health care were explored by Maganty et al.^17^ They queried primary care doctors who resided in rural areas for both barriers and strategies for addressing these barriers. There were five themes that developed as solutions and were as follows: subsidize services, establish mobile clinics for specialty care, increase utilization of telehealth, improve ancillary patient support, and increase utilization of APPs. Our tele-cystoscopy model employs three of the five themes to address a need for surgical subspecialty care. This is an issue facing general surgery and many surgical subspecialties not just urology.^18^ The broad skill set of the traditional rural surgeon crossed multiple disciplines such as orthopedics, urology, critical care, cardiac and gastrointestinal. With advances in technology and pharmaceutical development coupled with increasing call and work life balance, the solo do-it-all surgeon is a rare entity. There are concerns that residency with such sub-specialization does not prepare a surgeon for this type of practice. The solution of just training “more rural surgeons or urologists” did not seem feasible. We felt that a mobile or satellite clinic was a better solution. Although most of these studies examine the provider perspective, few assessed how the patient felt about this model of care.

Our academic medical center includes the catchment area of rural Virginia and parts of Appalachia in Tennessee and West Virginia. We devised a three-pronged approach in rural clinics for patients presenting with hematuria or for bladder cancer surveillance comprised of technologic infrastructure for transmitting the endoscopic video, APP training and competence, and external validation of cancer identification that we termed tele-cystoscopy. We have successively validated each of these components but have not reported on the patient perspective.^5,19,20^ In our previous work, we noted extended driving times of 5–7 h to our center for diagnostic and surveillance cystoscopy and sought to assess the socio-economic impact and patient satisfaction of tele-cystoscopy for these patients.

## Methods

An IRB protocol was developed that emphasized the components of tele-cystoscopy including testing the quality of the technical infrastructure, training of APPs, noninferior outcomes compared to patients seen at the primary tertiary health center, patient safety, defined as adverse events, and subjective patient satisfaction with telehealth services.^5,19–22^

The research team was composed of an APP, video analyst, and urologist who traveled with the necessary equipment and supplies, and the nursing team at each site assisted in patient preparation and high-level disinfectant of the equipment. The video analyst ensured the academic urologist was logged on and the telecommunication link was intact during the actual procedures. The APP had undergone training broadly based on the British Association of Urologic Nursing cystoscopic training manual augmented with a living cadaver endoscopic experience, and in person proctoring until they had reached proficiency as defined by cystoscopy checklist.^5^

Rural clinics referred patients for the procedure and the study determined if the procedure was warranted at the clinic visit. The inclusion criteria were quite broad and consisted of the clinical indication of hematuria (microscopic or gross), lower urinary tract symptoms, and prior history of bladder cancer. Patients with incontinence alone were excluded. Patients had to agree and consent to participate. The protocol consented patients for dual sequential cystoscopy. This model had the patients’ consent to a standard-of-care cystoscopy performed by a board-certified urologist. This was followed by the APP performing a second cystoscopy called tele-cystoscopy that was transmitted in real time to the remote urologist interpreting the images. The APP was blinded to the findings of the first cystoscopy as one aspect of the project was diagnostic concordance.^22^

Tele-cystoscopy patient appointments occurred in the University of Virginia Health System, Charlottesville, VA, service area; however, the remote clinics were much closer to their primary residence. We partnered with a hospital in Culpeper, Virginia, and two rural clinics in Wise, Virginia and Saltville, Virginia. The counties surrounding the two rural clinics have locum tenems urologists intermittently. Bristol, Tennessee, and Christiansburg, Virginia are where the two closest urology practices are located, and these locations will be 1–3 hour away for most residents. These practices may limit access based on insurance type. Upon completion of the procedures, patients filled out a questionnaire that contained both subjective and objective health and socioeconomic-related questions as well as qualitative comments. The questionnaire inquired about gender, race, ethnicity, age, and insurance as well as travel burdens including travel time saved and time off work.

Patients also filled out the Client Satisfaction Questionnaire (CSQ-8), a validated survey that is used in a variety of industries as an indicator of satisfaction. A score of 32 indicates the highest satisfaction with the service provided. Participants filled out a form on the day of service at the remote clinic.^23^ The patients were called a week later to identify if any adverse events had occurred.

Statistical averages were used to calculate the total travel distance and time saved, the cost of gas, and the time saved from work. Patient zip code information was correlated with the Distressed Communities Index (DCI) which combines economic factors including income, education, housing, and employment to measure economic well-being into a score from 1 to 100 and stratifies respondents into quintiles DCI 1–5 (1: prosperous, 2: comfortable, 3: mid-tier, 4: at-risk, and 5: distressed).^24^

## Results

**FIG. 1.**
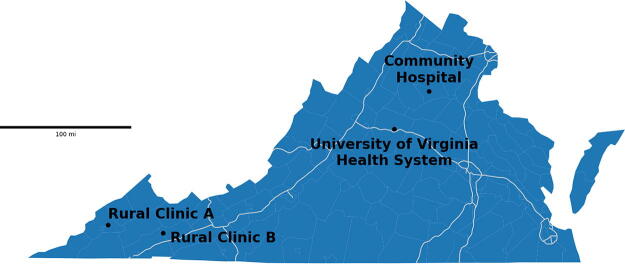
Geographic distribution of rural clinics, community hospital, and the University of Virginia Health System, Charlottesville, VA. The white lines represent interstates in the state of Virginia and the scale bar depicts 100 miles on the map.

The locations of the two rural clinics and the community hospital are shown geographically in relation to the University of Virginia Health System, Charlottesville, VA, in Figure 1. As a frame of reference, the one-way driving distance from Rural Clinic A is on average 5 h on Highway 81 which has a 70 mile/h speed limit while the distance from Rural Clinic B is 3.5 h.

Forty-eight patients participated in the study over the course of 4 years. The average age, race, ethnicity, gender, and insurance status are depicted in Table 1. Two patients were seen at the community hospital whereas the remaining 46 were seen at the rural clinics. This was a young population with an average age of 55 and a slight predominance of females to males. Twenty-seven percent had Medicare whereas less than 19% had private insurance.

**Table 1.** Patient Characteristics

Demographics	*N* (%)
Age (mean [range])	55.6 [30, 89]
Gender	
Male	21 (43.8%)
Female	27 (56.3%)
Race	
African American/Black	2 (4.2%)
Caucasian/White	42 (87.5%)
Other	1 (2.1%)
Unknown	3 (6.3%)
Ethnicity	
Hispanic	1 (2.1%)
Non-Hispanic	44 (91.7%)
Unknown	3 (6.3%)
Insurance	
Uninsured	13 (27.1%)
Private	9 (18.8%)
Medicare	13 (27.1%)
Medicaid	10 (21.1%)
Medicare/Medicaid	2 (4.2%)
Unknown	1 (2.1%)

Table 2 lists the perceived benefits from the patient’s perspective. In terms of travel burdens, 27% would have traveled 3–5 h for the same service, whereas 8% would have had over 5 h of travel time. While most of the patients were retired or not working (80%), the remaining 20% would have missed a half-day or full day of employment.

**Table 2.** Perceived Patient Benefits

	*N* (%)
Travel time saved	
<30 min	7 (14.6%)
30 min–1 h	6 (12.5%)
1–3 h	4 (8.3%)
3–5 h	13 (27.1%)
5–7 h	2 (4.2%)
7 + h	2 (4.2%)
Time off Work	
None, day off	17 (35.4%)
None, unemployed/retired	21 (43.8%)
<4 h	5 (10.4%)
4–8 h	2 (4.2%)
>8 h	2 (4.2%)
Help with transportation	
Yes	6 (12.4%)
No	42 (87.5%)

For DCI quintiles, 82% of our patients resided in a distressed county, 2% were in an at-risk county, and 14% were in a mid-tier county. Only 2% were in a prosperous county.

Table 3 quantifies the mileage difference between the rural clinics and the University of Virginia Health System, Charlottesville, VA. The average round trip distance and travel time to the academic site from patients seen at the rural clinics were 540 miles and 524 min (median 554 miles and 542 min), respectively. On average, the tele-cystoscopy clinic saved 489 miles and 452 min of travel time (median 513 miles and 451 min). For the two patients seen at the community hospital, they saved an average of 58 miles and 59 min of travel time round trip being seen via the tele-cystoscopy clinic.

**Table 3.** Patient Travel Characteristics for the Patients Seen for Tele-Cystoscopy at the Rural Clinics

	Average		Median	Standard deviation	Minimum	Maximum
	One way	Round trip				
Travel Time to Academic Site (min)	262	524	271	36.4	198	320
Distance to Academic Site (miles)	270	5404	277	30.5	209	317
Travel Time to Rural Clinic (min)	36	72	29	25.9	8	96
Distance to Rural Clinic (miles)	25	50	18	21.9	4	81
Total Time Saved (round trip)		452 min				
Total Distance Saved (round trip)		489 miles				

Note: All statistics are presented for one direction of travel unless otherwise specified.

**FIG. 2.**
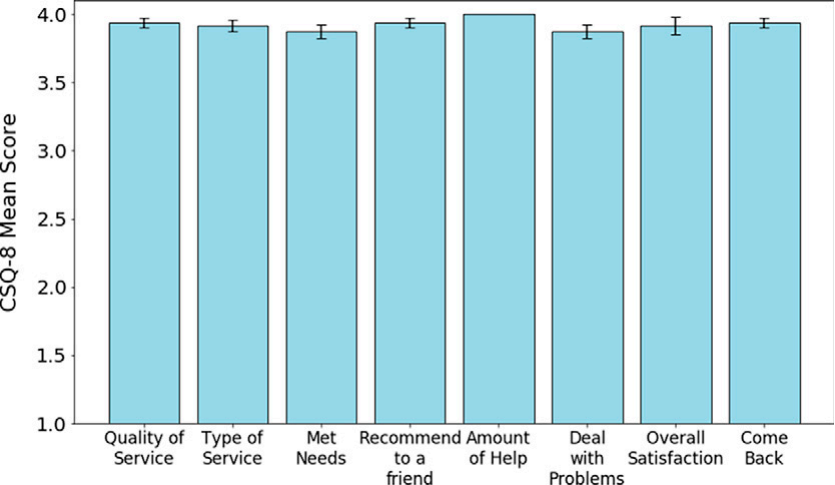
Average scores for each component of the CSQ-8. Each of the eight components had a maximum possible score of 4, for a maximum possible overall score of 32, and a minimum of 1 for each possible score. The median score for each component was 4. CSQ-8, Client Satisfaction Questionnaire.

Satisfaction scores for the tele-cystoscopy clinic are in Figure 2. Forty-seven patients completed CSQ-8 surveys with a mean score of 31.38 (±1.35) with a median score of 32. One patient had burning with urination that was treated with 3 days of antibiotics without a confirmatory culture.

### Qualitative comments

**FIG. 3.**
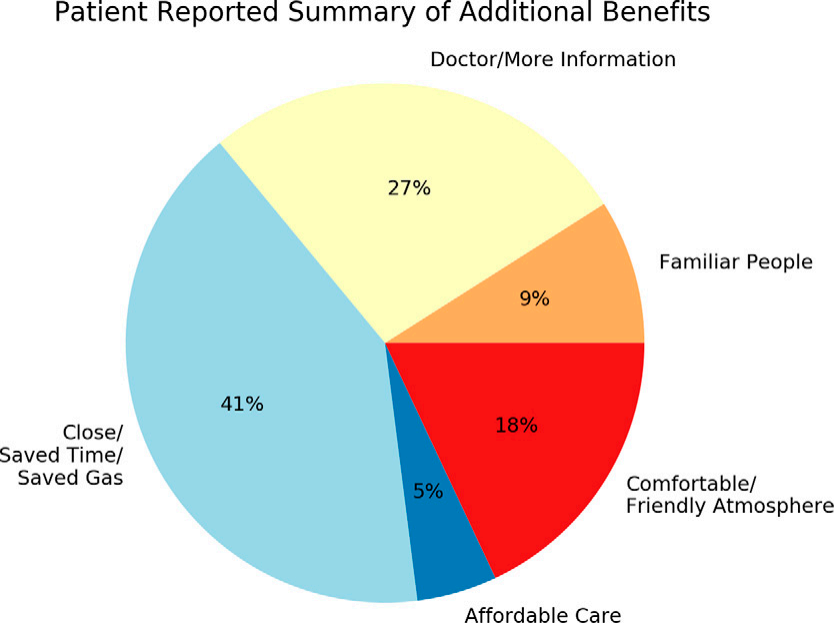
Summary of additional patient-reported benefits of tele-cystoscopy.

Figure 3 depicts a summary of the qualitative survey responses from patients. The most frequent responses were about convenience in terms of distance traveled, time saved, or saved gasoline expenses. Patients also noted the convenience of having a physician with more expertise about bladder cancer.

## Discussion

This novel application of telemedicine was overwhelmingly appreciated by our patients in rural and underserved areas. The DCI rating of the majority of our patients speaks to the underserved nature of these communities. Given that most of the patients surveyed were economically distressed, the added burden of a significant distance traveled (up to 8 h) for urologic care does represent a substantial hardship. On average, the tele-cystoscopy clinic saved patients 7 h of travel time, almost 1 standard workday. Utilizing telemedicine and urology trained APPs, this unique clinic provides a model of how to leverage current resources in anticipation of urology workplace shortages.

The impact of forthcoming urology workforce shortages is particularly felt in rural and underserved areas. In 2022, 62% of American counties had no practicing urologists and up to 35% of new patients in rural areas waited 4 weeks or longer to schedule an initial visit.^11,25^ A recent needs assessment conducted by the University of Virginia Cancer Center [2019 Community Health Assessment Survey collected by the UVACCC (P30CA044579-27S5 Population Health Supplement)] echoed this point, with 21% of patients delaying medical care because they “couldn’t get an appointment soon enough.” Importantly, 95% of patients in southwest Virginia (where the two rural clinics in our study were located) have a regular medical provider they see when they are sick or need medical advice. Among these patients, 92% are able to travel to their medical provider from their home within 30 min. The tele-cystoscopy model provides a comparable level of access to specialty care as these rural patients have for primary care visits. In addition, transportation to medical care in the southwest Virginia region was “not at all difficult” for 88.5% of the population, with a further 10% finding transportation “not too difficult.” This further supports the need for specialty care to be brought closer to the patient. Most of these patients did not have reliable access to urologic care as the largest group in the area is across the border in Tennessee and often would not take patients with Virginia Medicaid or no insurance. Similarly, locum tenens are inconsistently providing care in the region.

### Practical use of telehealth in urology

Telehealth has been reported as economically feasible across several specialties while also reducing burden on patient travel distance, patient wait times, and improving rural patient access. Urology as a specialty has been keen to adopt telehealth practices that range in function and structure. A review on the use of telehealth in urology found 24 articles in the past 10 years that cited specific uses of telemedicine technology and found that the most common application of telehealth was follow-up care with patients after procedures or visits but noted that other unique examples like our novel application of tele-cystoscopy that improved patient access to care.^26^

Patient satisfaction and ease of use are important factors that impact adherence to follow-up which is a tenet of NMIBC surveillance. AUA guidelines for NMIBC recommend surveillance based on patient risk with researchers noting varying levels of compliance. In Iowa, a largely rural state, Tobert et al. found that shorter distance to urology providers was associated with more screening cystoscopies and that patients living more than 30 miles from a urology office received 1 fewer cystoscopy than patients living less than 10 miles from a urology office. They also noted the inherent trade-off in using outreach clinics which were more convenient for patient follow-up but lacked diagnostic and treatment resources. Our patients overwhelmingly noted the convenience of time and distance saved through participation in the tele-cystoscopy clinic. The tele-cystoscopy model alleviates this resource disparity and allows patients and providers to adhere to guidelines without compromising treatment.^27^

The continued success of telehealth has been built upon marked innovation and advancement of information and communication technologies, namely reduction in cost and increase in the availability of high-speed internet access, and a necessity. Additional growth in the adoption and use of telehealth was a direct result of the COVID-19 pandemic in which health care providers and patients searched for contactless ways to maintain regular appointments.^2^

Even with proven successes, there are still barriers to telehealth adoption in the field of urology. Naik et al. note that while there is great potential and a need for telehealth to act as a medium to provide continuous high-value care to a wide variety of patients, hurdles exist at legislative and technological levels that will hinder progress until remedied. Notably, they argue for reforms that relate to societal guidelines, patient awareness, and provider and patient comfort with telehealth technologies.^7^ Policy and legislation changes needed to alter the current trajectory of the urologic workforce and address systemic issues like this are difficult to implement and can take years for differences to be felt. Thus, there is a great need for optimization and implementation of current technology and procedures to address systemic issues that will be mutually beneficial for patients and providers alike, especially those in rural areas. Although telemedicine has been suggested to have benefits to the nearly 20% of Americans living in rural areas, studies show that certain patients including patients that live in rural areas, are older, nonwhite, and of lower socioeconomic status were less likely to use telemedicine.^28^

Our current study addressed patient satisfaction with a novel application of telemedicine and its implications for bladder cancer diagnosis and surveillance in rural areas. As with all studies, we have limitations. Our sample size is small and we cannot with any confidence identify if subsets of patient were more satisfied with tele-cystoscopy than others. Similarly, we cannot associate any demographic variable with outcomes with any degree of certainty. The patients tend to be very grateful for our willingness to travel to them and we imagine there is an unconscious bias to review us favorably. Hence, the satisfaction rates may be artificially elevated. Nonetheless, we found that patients were overwhelmingly satisfied with the improved drive times to appointments and felt that their medical care was improved by having access to physicians with more expertise in bladder cancer.

## Conclusion

The forthcoming urology workforce shortage necessitates creative and complex problem-solving at all governmental and health-system levels. Applications of telemedicine as we have described here do require legislative support for infrastructure and implementation but have the benefit of leveraging current legislative and community stakeholders to expand access to rural health care. Here, we describe the results of the pivotal pilot study done primarily in rural Appalachian communities that provide further evidence in favor of the implementation of telehealth services in urology to address bladder cancer surveillance. Our evidence primarily points to reductions in patient travel distances, time saved by utilizing telehealth services, patient satisfaction with service, noninferiority of telehealth services rendered, and guidelines that would ease implementation for health care systems seeking to utilize a telehealth model.

## Authors’ Contributions

A.O.K.: Formal Analysis, Data Curation, Writing—Original Draft. W.H.: Formal Analysis, Data Curation, Writing—Original Draft. T.S.: Conceptualization, Resources, Writing—Review and Editing. S.H.C.: Conceptualization, Writing—Review and Editing, Supervision. T.L.K.: Conceptualization, Resources, Writing—Review and Editing, Supervision, Funding Acquisition. J.M.L.: Conceptualization, Formal Analysis, Data Curation, Writing—Review and Editing, Visualization, Supervision, Funding Acquisition.

## Abbreviations Used

APP: advanced practice providers
AUA: American Urological Association
CSQ-8: Client Satisfaction Questionnaire
DCI: Distressed Communities Index
NMBIC: nonmuscle invasive bladder cancer
